# Hot Under the Collar: Humidity, Heat, and Heart Rhythms

**DOI:** 10.1016/j.jacadv.2024.101460

**Published:** 2024-12-13

**Authors:** Sadeer Al-Kindi, Jad Ardakani, Kai Chen

**Affiliations:** a DeBakey Heart and Vascular Center, Houston Methodist, Houston, Texas, USA; b Center for Health and Nature, Houston, Texas, USA; c Department of Environmental Health Sciences, Yale School of Public Health, New Haven, Connecticut, USA; d Yale Center for Climate Change and Health, Yale University, New Haven, Connecticut, USA

**Keywords:** arrhythmia, environment, heat, humidity

**Figure:**
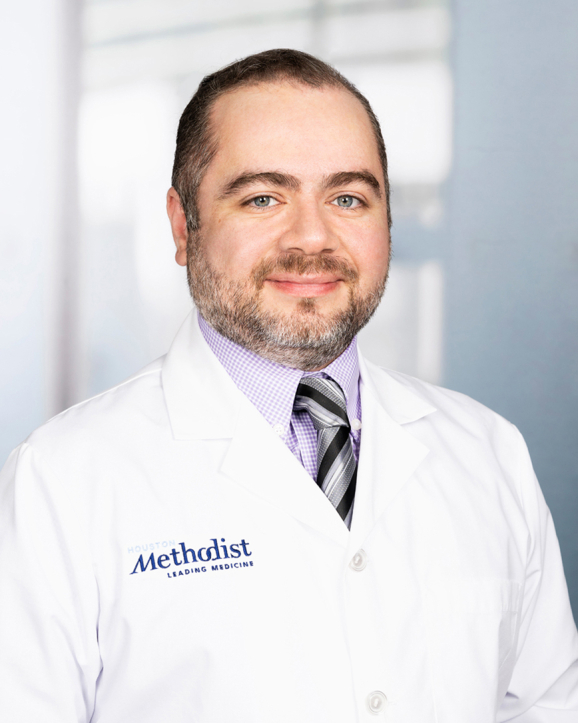

As global environmental conditions continue to shift, their impact on public health is becoming increasingly evident. There is growing attention on how external factors (ie, the environmental exposome—encompassing the totality of an individual's environmental exposures) influence cardiovascular health.^1^, ^2^, ^3^ Research consistently demonstrates that rising temperatures and air pollution contribute to higher rates of cardiovascular diseases and mortality.^4^ Yet, the effects of other factors, such as humidity, on cardiovascular health are not as well understood. Additionally, identifying patients who are most susceptible to these environmental influences requires further investigation. Understanding the intricate relationships between these environmental variables, cardiovascular health, and susceptible populations is essential. This knowledge will facilitate the development of effective public health interventions and enhance patient outcomes in an era characterized by rapid environmental change.

In this issue of *JACC: Advances*, Keeler et al^5^ offer crucial insights into how climate factors specifically affect patients with cardiovascular disease. This longitudinal study, spanning from 2010 to 2021, analyzed the geocoded health records of 5,944 patients with implanted cardiac devices and linked weather data to assess the impacts of extreme temperature and humidity on the incidence of ventricular arrhythmias. Key findings from this comprehensive analysis revealed that extreme humidity was significantly associated with the risk of ventricular arrhythmias. Notably, on days where relative humidity reached or exceeded the 95th percentile, there was a 23% increase in the risk of arrhythmias over a 7-day period (from current exposure day to 6 days postexposure). In contrast, extreme temperature alone was not found to be associated directly with an increased risk, suggesting that the high moisture content in the air plays a more critical role in affecting cardiac function than temperature alone.

Importantly, the study revealed that susceptibility to ventricular arrhythmias due to extreme humidity varies significantly across different demographic and clinical groups. Notably, certain populations showed heightened vulnerability: males, older adults (particularly those aged 67-75), and individuals with pre-existing cardiometabolic conditions such as coronary artery disease, heart failure, diabetes, and hypertension. These groups exhibited the most pronounced risk increases following exposure to high humidity levels. Additionally, socioeconomic factors played a crucial role, with communities experiencing high socioeconomic deprivation and higher income inequality also showing increased susceptibility to humidity. This indicates that both biological and social determinants of health critically influence how individuals and communities respond to environmental stressors like extreme humidity, underscoring the need for targeted public health interventions and policies to protect these at-risk populations.

The study boasts several strengths that lend credibility and depth to its findings such as relatively large cohort size and long study period. Moreover, the study utilized a case time-series design which significantly reduces confounding by allowing each participant to serve as their own control. This design effectively controls for non-time-varying characteristics, such as long-term health behaviors or clinical histories, ensuring that the findings are attributable to the weather conditions rather than underlying patient differences. Despite its strengths, the study also has several limitations that must be considered. As a retrospective observational study, it cannot establish causality making it difficult to definitively conclude that extreme humidity causes increased arrhythmia risk without potential unknown confounders influencing the results. Additionally, the study focuses solely on relative humidity and does not account for other forms of humidity measurement (eg, absolute humidity, dew point) that might offer different insights. Compared to relative humidity that is influenced by temperature, mass-based measures such as absolute humidity could directly affect sweat evaporation and is recommended for epidemiological studies.^6^ The geographical focus on North Carolina also limits the generalizability of the findings to other regions with different climate conditions. Furthermore, the study did not account for air pollution, a known trigger for cardiac arrhythmias,^7^ which might confound the relationship between humidity and arrhythmias. The lack of adjustment for all possible time-varying confounders, like acute stress or behavioral changes (eg, sleep) in response to weather conditions, also poses potential limitations to the findings' accuracy.

The findings of this study contribute to a growing body of research examining the link between climate factors and cardiac health. While some studies have investigated the associations between ambient temperature and arrhythmia, humidity has received less attention.^8^, ^9^, ^10^ It is important to note that only one^8^ of these 3 studies have examined humidity as a confounder. One prior small study of 84 patients with implantable cardioverter-defibrillators in eastern Massachusetts showed that lower temperature and lower absolute humidity were associated with increased ventricular arrhythmia.^11^

By focusing only on the warm seasons, this study potentially excluded variations in temperature and humidity that occur in cooler seasons, which might influence the incidence of arrhythmias differently. This seasonal limitation means that the colder months, which could provide contrasting data on the effects of lower temperatures on ventricular arrhythmias, were not considered. Such a focus restricts the ability to fully understand the impact of a broader range of temperature variations throughout the year, possibly skewing the results to only reflect the conditions most prevalent in warmer months. This limitation is crucial for interpreting the study findings, as it suggests that additional research might be needed to explore how cooler weather conditions affect arrhythmia risks in patients with cardiac devices.

The mechanisms linking high temperatures, especially when combined with high humidity, with cardiac arrhythmias encompass several physiological processes.^12^ High ambient temperatures challenge the body's homeostatic mechanisms, leading to increased cardiac output, dehydration, and elevated heart rates, which collectively can precipitate arrhythmic events. Furthermore, heat influences electrolyte balance and myocardial ion channel function, crucial for maintaining heart rhythm, and exacerbates inflammation and oxidative stress—known factors in cardiovascular disease pathology.^12^ Humidity exacerbates these temperature effects by impairing the body's cooling mechanisms, leading to an even greater physiological burden. High humidity prevents efficient sweat evaporation, a key cooling process, resulting in increased internal body temperatures and cardiovascular strain.^13^ This can manifest as increased sympathetic tone, heart rate and altered blood pressure, heightening arrhythmia risks, especially in individuals with cardiovascular vulnerabilities (including those with implanted cardiac devices).

This study underscores the urgent need for further research to explore the relationship between climate factors and cardiovascular health across diverse climates and larger populations. Expanding this research globally would help validate the findings and refine our understanding of how different environments influence cardiovascular risks. Additionally, there is a pressing need for the development of advanced predictive tools that incorporate real-time environmental data. Such tools would revolutionize the management of cardiovascular diseases by enabling personalized patient care plans that adapt to daily environmental conditions. Moreover, these findings should catalyze the integration of environmental health factors into public health strategies and urban planning. Policies aimed at increasing urban green spaces could mitigate some impacts of high temperatures and humidity, thereby reducing the public health burden of heat-related cardiovascular incidents. Health care providers also need to consider environmental risk factors in their clinical assessments and patient education strategies, particularly for vulnerable populations with pre-existing cardiovascular conditions.

## Funding support and author disclosures

The authors have reported that they have no relationships relevant to the contents of this paper to disclose.

## References

[1] Motairek I., Makhlouf M.H., Rajagopalan S., Al-Kindi S. (2023). The exposome and cardiovascular health. Can J Cardiol.

[2] Al-Kindi S.G., Brook R.D., Biswal S., Rajagopalan S. (2020). Environmental determinants of cardiovascular disease: lessons learned from air pollution. Nat Rev Cardiol.

[3] Wass S.Y., Hahad O., Asad Z. (2024). Environmental exposome and atrial fibrillation: emerging evidence and future directions. Circ Res.

[4] Khraishah H., Alahmad B., Ostergard R.L. (2022). Climate change and cardiovascular disease: implications for global health. Nat Rev Cardiol.

[5] Keeler C., Cleland S.E., Hill K.L. (2024). Effects of extreme humidity and heat on ventricular arrhythmia risk in patients with cardiac devices. JACC Adv.

[6] Baldwin J.W., Benmarhnia T., Ebi K.L., Jay O., Lutsko N.J., Vanos J.K. (2023). Humidity’s role in heat-related health outcomes: a heated debate. Environ Health Perspect.

[7] Pallikadavath S., Vali Z., Patel R. (2022). The influence of environmental air pollution on ventricular arrhythmias: a scoping review. Curr Cardiol Rev.

[8] Li M., Shaw B.A., Zhang W., Vásquez E., Lin S. (2019). Impact of extremely hot days on emergency department visits for cardiovascular disease among older adults in New York state. Int J Environ Res Publ Health.

[9] Zanobetti A., Coull B.A., Kloog I., Sparrow D., Vokonas P.S., Gold D.R., Schwartz J. (2017). Fine-scale spatial and temporal variation in temperature and arrhythmia episodes in the VA Normative Aging Study. J Air Waste Manag Assoc.

[10] McGuinn L., Hajat S., Wilkinson P., Armstrong B., Anderson H.R., Monk V., Harrison R. (2013). Ambient temperature and activation of implantable cardioverter defibrillators. Int J Biometeorol.

[11] Nguyen J.L., Laden F., Link M.S., Schwartz J., Luttmann-Gibson H., Dockery D.W. (2015). Weather and triggering of ventricular arrhythmias in patients with implantable cardioverter-defibrillators. J Expo Sci Environ Epidemiol.

[12] Crandall C.G., González-Alonso J. (2010). Cardiovascular function in the heat-stressed human. Acta Physiol.

[13] Davis R.E., McGregor G.R., Enfield K.B. (2016). Humidity: a review and primer on atmospheric moisture and human health. Environ Res.

